# Trends and advances in *Leptospira*, a bibliometric analysis

**DOI:** 10.3389/fmicb.2024.1514738

**Published:** 2025-01-08

**Authors:** Wei Wang, Yamin Gao, Jianyu Ji, Zhai Huang, Bin Xiong, Shulin Xiang

**Affiliations:** ^1^Department of Intensive Care Unit, The Peoples Hospital of Guangxi Zhuang Autonomous Region, Nanning, Guangxi, China; ^2^Research Center of Communicable and Severe Diseases, Guangxi Academy of Medical Sciences, Nanning, Guangxi, China; ^3^Guangxi Health Commission Key Laboratory of Diagnosis and Treatment of Acute Respiratory Distress Syndrome, Nanning, Guangxi, China; ^4^Guangxi Academy of Medical Sciences, Nanning, Guangxi, China

**Keywords:** *Leptospira*, bibliometric analysis, research hotspots, trends, Web of Science Core Collection

## Abstract

**Background:**

Leptospirosis is an acute zoonotic disease caused by pathogenic *Leptospira*, primarily transmitted to humans through contact with water or soil contaminated by the bacteria. It is globally distributed, with heightened prevalence in tropical regions. While prior studies have examined the pathophysiology, epidemiology, and risk factors of leptospirosis, few have explored trends and emerging topics in the field. This study applies bibliometric analysis to generate a visual knowledge map, identifying research hotspots and forecasting future trends in leptospirosis investigations.

**Methods:**

Data were extracted from the Web of Science Core Collection (WOSCC), encompassing all publications up to May 1, 2024. CiteSpace and VOSViewer software were used to analyze annual publication trends, as well as contributions from countries, institutions, journals, authors, references, and keywords, thereby pinpointing current research priorities and potential future directions.

**Results:**

A total of 5,244 articles were included, sourced from 4,716 institutions, 955 journals, and 156 countries or regions. The United States led with 1,315 publications and had the most significant influence in the field. “PLOS Neglected Tropical Diseases” published the highest number of articles (166), while “Infection and Immunity” garnered the most citations (6,591). Prominent research areas included restriction endonucleases, monoclonal antibodies, outer membrane proteins, water environments, detection methods, and antimicrobial agents. Research focus has shifted from early genomic and antigenic studies to investigations into outer membrane protein functions and environmental persistence, culminating in recent advances in molecular mechanisms and diagnostic technology development.

**Conclusion:**

This bibliometric analysis provides a comprehensive snapshot of leptospirosis research, emphasizing collaborations and impact among authors, countries, institutions, and journals. It offers valuable insights into ongoing trends and serves as a reference for future collaboration and research opportunities in the field.

## 1 Introduction

Pathogenic *Leptospira* are primarily transmitted *via* rodents, pigs, cattle, and sheep, with infection primarily originating from urine excreted by these carrier animals into water sources (Dara, 2019). Humans become infected upon contact with contaminated water, particularly when the bacteria penetrate through the skin or mucous membranes, especially *via* broken skin. Once inside the body, *Leptospira* disseminates through the bloodstream, impacting various organs and leading to multi-organ dysfunction (Mathieu, 2017). Clinical manifestations of leptospirosis vary, ranging from mild influenza-like symptoms to severe impairment of vital organs such as the lungs, liver, and kidneys. In severe cases, patients may experience hypotensive shock, pulmonary hemorrhage, acute liver and kidney failure, and multi-organ dysfunction syndrome, with mortality rates reaching up to 50% (Ajay et al., 2003; Eddy et al., 2005).

Leptospirosis prevalence is influenced by a complex interaction of pathogen, host, and environmental factors, compounded by geographical, natural, and socio-economic conditions that heighten the risk of outbreaks and human transmission. Despite its life-threatening nature, the pathogenesis of leptospirosis remains incompletely understood, and global incidence and mortality estimates remain unclear (Federico et al., 2015). Furthermore, limited awareness among clinicians and public health professionals has hindered effective prevention and control measures.

Bibliometric analysis serves as a valuable tool for assessing the status of research fields, identifying scientific achievements, and pinpointing emerging hotspots (Wei et al., 2024). This approach has proven beneficial in various biomedical fields, including inflammation, immunity, and cancer (Peixiang et al., 2024; Qi et al., 2024; Xiang'an et al., 2024; Ziye et al., 2024). Although leptospirosis has gained attention in recent years, specific research trends and emerging focus areas remain unclear. To address this gap, a bibliometric analysis of leptospirosis-related literature from the WOSCC, spanning the last 30 years, was conducted. Specialized bibliometric software was used to assess contributions from countries, institutions, journals, authors, references, and keywords, identifying the most influential entities. Additionally, outbreak-related references were retrieved, and key thematic clusters in leptospirosis research were identified. The goal was to construct a visual model to evaluate current research trends, track the evolution of the research landscape, and predict future directions. This study provides a comprehensive visual knowledge map and valuable insights into leptospirosis, offering a critical reference for researchers and guiding future research initiatives.

## 2 Methods

### 2.1 Retrieval strategy and data collection

**Figure 1:**
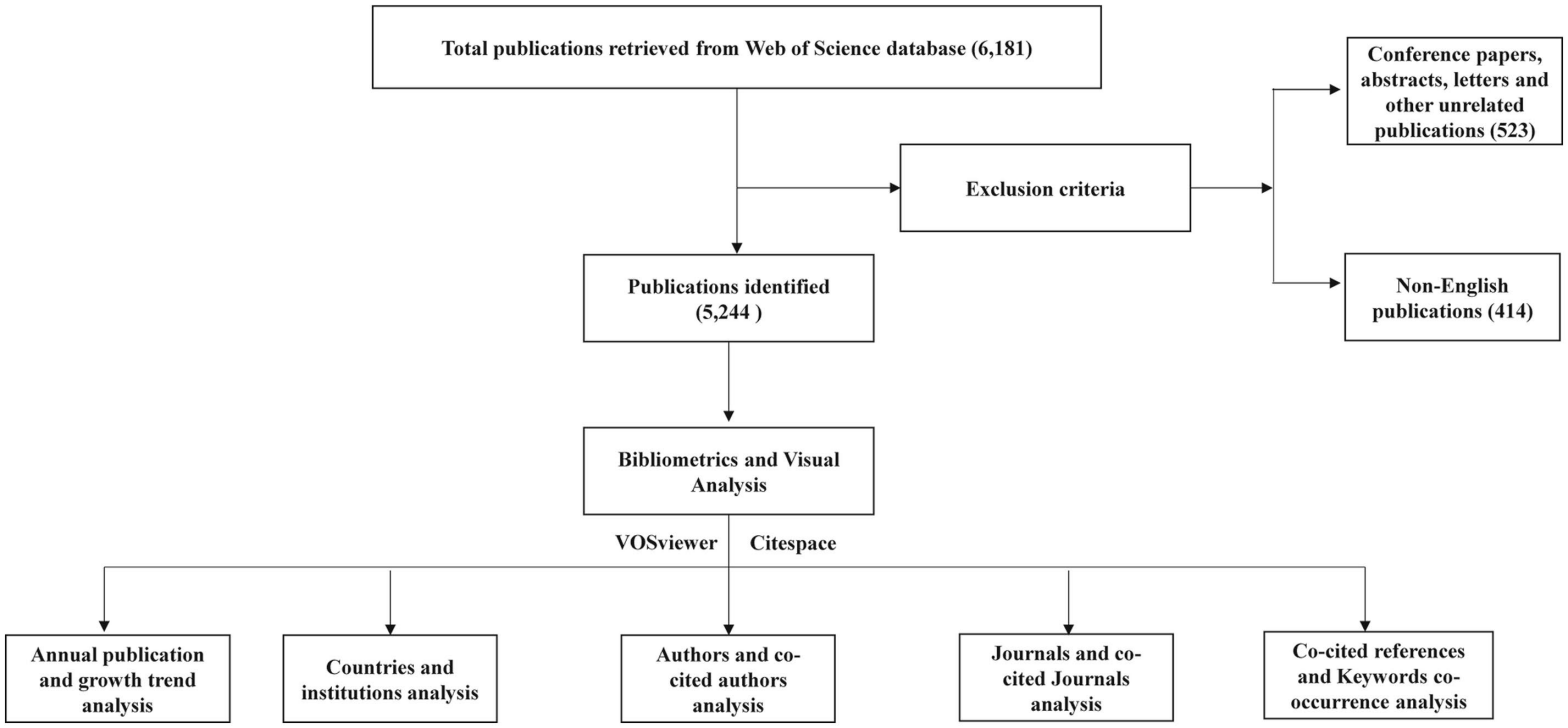
Flow chart of bibliometric analysis for *Leptospira* related studies.

The WOSCC was selected as the data source for this study due to its global influence, authority, and comprehensive nature, providing extensive citation data across multiple disciplines. This feature makes WOSCC particularly advantageous for multidisciplinary and international bibliometric analyses (Christian et al., 2024). Our data retrieval strategy used the following search terms: (TS = (Leptospirosis)) OR (TS = (*Leptospira*)), with a search period up to May 1, 2024. This search yielded a total of 6,181 relevant publications. Two authors independently screened the literature according to predefined inclusion and exclusion criteria, focusing on articles and reviews published in English. Initial screening involved examining titles and abstracts, which led to the exclusion of 523 documents, including conference papers, abstracts, letters, and other non-relevant document types. Additionally, 414 non-English publications were excluded. The final dataset consisted of 5,244 articles (Figure 1). The data was then exported in both full-record plain text and tab-delimited file formats, containing metadata such as titles, authors, abstracts, keywords, nationalities, research institutions, and citation information, preparing it for further analysis.

### 2.2 Statistical methods

For the bibliometric analysis, we used VOSviewer 1.6.19 (Leiden University, Netherlands) to explore co-cited articles, keywords, countries/regions, institutions, journals, authors, and references. CiteSpace 6.1.R6 (Drexel University, Pennsylvania, USA) was employed for cluster analysis of co-occurring keywords and outbreak term analysis. The main parameters of our analysis included countries, institutions, journals, authors, references, and keywords. To visualize the trends in publication and citation numbers over time, we utilized Microsoft Office Excel 2013 to create line graphs.

### 2.3 Data processing

The dataset, exported in RefWorks format, was initially named “download_.” We performed data de-duplication and conversion using CiteSpace 6.3.R1. Following data organization, the information was imported into bibliometric software for analysis. For institutional collaboration, network analysis, and keyword co-occurrence analysis, we employed VOSviewer 1.6.18. Further analysis using CiteSpace 6.3.R1 included author collaboration, network analysis, keyword clustering, and timeline analysis to provide a comprehensive overview of the current research landscape in leptospirosis. CiteSpace was configured with a time span from 1967 to 2024, setting “Years Per Slice” to one year. Additionally, synonymous keywords and institutions were merged to ensure consistency in the analysis. In VOSviewer, the “Co-occurrence” option was selected from the “Type of analysis” menu for in-depth keyword analysis.

## 3 Results

### 3.1 Annual publication summary

**Figure 2:**
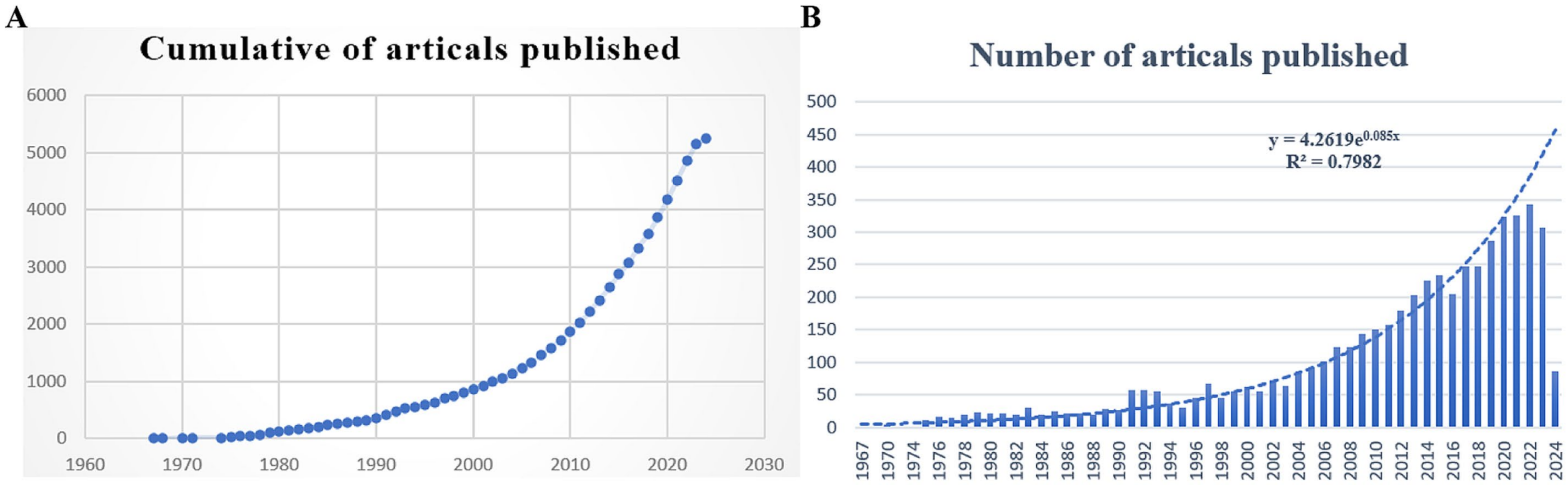
Number of *Leptospira* related articles published in Web of Science from 1967 to 2024. **(A)** Cumulative number of articles published; **(B)** annual number of articles published and article publication trends.

Our analysis covered 5,244 papers published between 1967 and 2024, comprising 4,922 articles and 322 reviews. As illustrated in Figure 2, the annual number of publications and citations related to leptospirosis shows a consistent upward trend. Research on leptospirosis began in 1967, marked by E. Shenberg’s development of a protein-free medium for culturing pathogenic *Leptospira* (Shenberg, 1967). For the next 25 years, the field experienced slow growth, with an average of about 20 articles published annually. This rate accelerated significantly in 1991, peaking in 2022 with 342 articles. Predictive algorithms indicate a continuing increase in research output in the coming years, suggesting that leptospirosis is gaining attention as a research hotspot globally.

### 3.2 Country/region analysis

**Figure 3:**
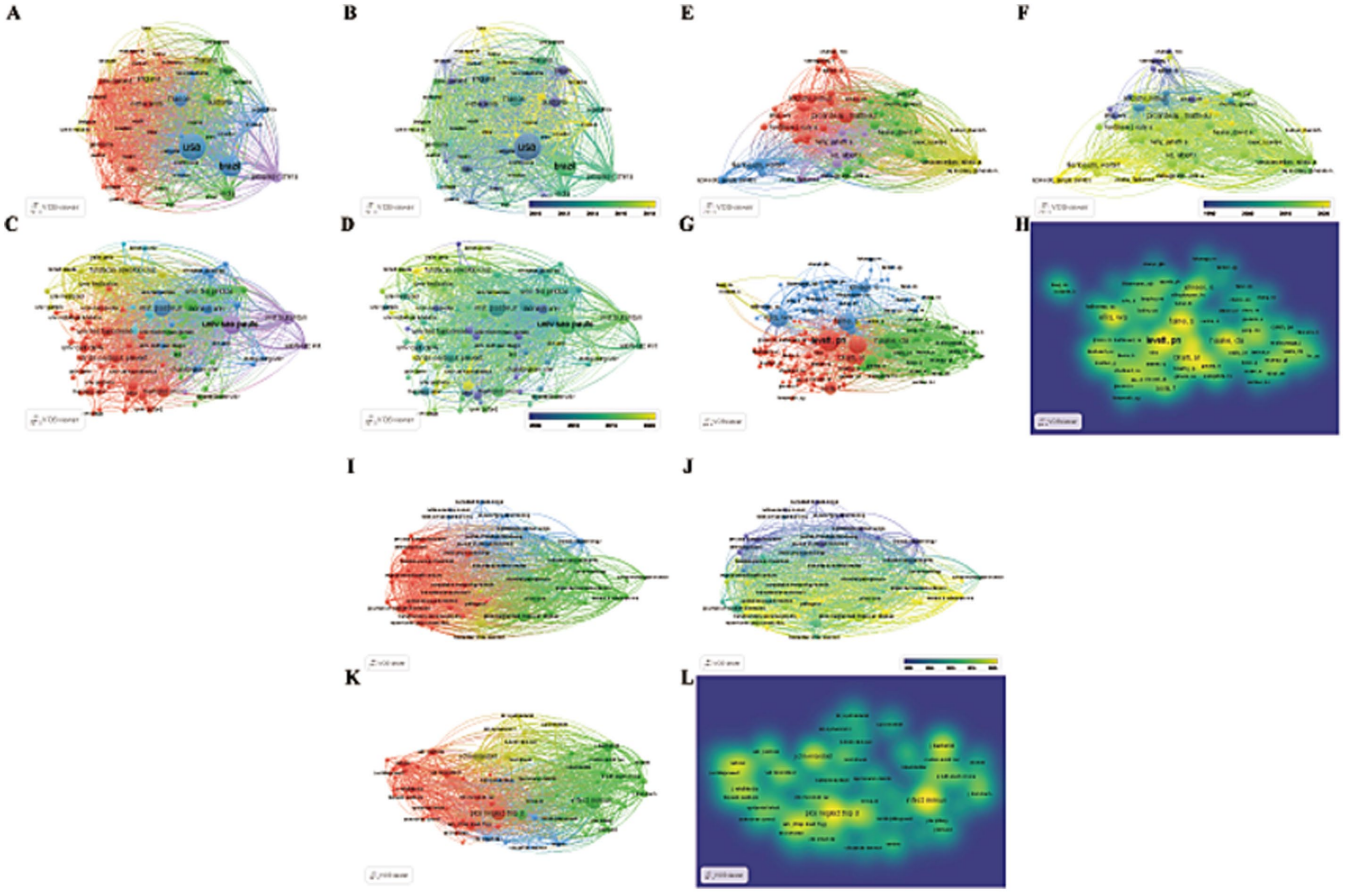
Visual maps created by VOSviewer. **(A,B)**: countries/regions; **(C,D)**: institutions; **(E,F)**: authors; **(G,H)**: co-cited authors; **(I,J)**: journals; **(K,L)**: co-cited journals. **(A,C,E,G,I,K)** Different color groups in the figure represent the cooperative relationship between various indicators. **(B,D,F,H,J,L)** Different color groups in the figure represent the time points at which various indicators are discovered.

The analysis revealed that 4,716 institutions from 156 countries/regions have contributed literature on *Leptospira*. Visualizing countries with more than 20 published articles, we found that 53 countries meet this criterion (Figures 3A,B). Table 1 lists the top 10 countries and institutions based on publication volume. The United States leads with 1,315 publications and 47,695 citations, followed by Brazil (890 publications, 18,573 citations) and France (411 publications, 14,860 citations). India ranks fourth with 390 publications and 4,487 citations, while China is fifth with 311 publications and 6,137 citations. Together, these five countries contribute approximately 58.5% of the total publications. The strength and tone of network connections illustrate the degree of research collaboration between countries or regions. Nodes closer to red indicate stronger cooperation. The results indicate that the United States has the most robust international collaborations (total strength: 47,377), followed by Brazil (30,066) and France (23,174), forming a broad cooperative network. Although India and China are among the top five in terms of publication numbers, their influence in terms of total citations and Total Link Strength (TLS) rankings is comparatively lower, this indicates that India and China have limited influence in high-quality literature and hot research, and need to further enhance their academic value.

**Table 1:** Top 10 countries in terms of number of articles published.

Rank	Country	Documents	TC	AAC	TLS
1	USA	1,315	47,695	36.27	47,377
2	Brazil	890	18,573	20.87	30,066
3	France	411	14,860	36.16	23,174
4	India	390	4,487	11.51	7,906
5	China	311	6,137	19.73	10,327
6	Australia	257	9,953	38.73	13,948
7	England	237	5,149	21.73	8,220
8	Japan	220	4,211	19.14	6,727
9	Thailand	218	5,094	23.37	9,706
10	Netherlands	197	5,917	30.04	9,734

TC, Total citations; AAC, Average article citations; TLS, Total link strength.

### 3.3 Institutional analysis

A total of 4,716 institutions are engaged in leptospirosis research. According to VOSViewer results, 88 institutions have published more than 20 articles (Figures 3C,D; Table 2). The University of São Paulo leads with 256 papers, followed by the Pasteur Institute (230 papers) and Cornell University (137 papers). In terms of total citations, San Bortolo Hospital ranks first (2,073 citations), followed by the University of Pittsburgh (1,978 citations) and the University of Michigan (1,813 citations). The top ten institutions are primarily located in the United States (40%), Australia (20%), and Brazil (20%), highlighting their extensive research interest and strong influence in this field.

**Table 2:** Top 10 institutions in terms of number of articles published.

Organization	Country	Documents	TC	AAC	TLS
University of São Paulo	Brazil	256	5,664	22.13	11,254
Pasteur Institute	France	230	10,623	46.19	13,283
Cornell University	USA	137	8,033	58.64	9,933
Instituto Butantan	Brazil	130	2,821	21.70	8,545
Mahidol University	Thailand	124	2,840	22.90	4,309
University of California Los Angeles	USA	99	9,058	91.49	11,514
Federal University of Pelotas	Brazil	97	2,435	25.10	6,092
Universidade Federal Fluminense	Brazil	94	1975	21.01	2,807
Monash University	Austria	83	5,243	63.17	7,944
Centers for Disease Control and Prevention	USA	83	6,491	78.20	5,381

TC, Total citations; AAC, Average article citations; TLS, Total link strength.

### 3.4 Author and co-cited author analysis

Our analysis identified contributions from 5,918 authors in leptospirosis research. Key scholars driving research trends were pinpointed using VOSViewer (Figures 3E–H; Table 3). Mathieu Picardeau from France led in both publication volume (117 papers, 4.61%) and citations, followed by Walter Lilenbaum (89 papers, 3.17%). The highest total link strength (TLS) was attributed to Walter Lilenbaum (5,969), followed by Albert I. Ko (4,074) and Ben. Adler (3,995). In terms of average article citations (AAC), Ben. Adler (71.78), Albert I. Ko (63.60), and David A. Haake (61.71) ranked highest. Table 3 presents a timeline of prominent authors, with larger circles denoting significant contributions at various time points. Albert I. Ko, David A. Haake, and Ben. Adler are identified as key pioneers, both as citing and co-citing authors, suggesting their teams as key potential collaborators in this study (Supplementary Table S1).

**Table 3:** Top 10 authors in terms of number of articles published.

Rank	Author	Country	Documents	TC	AAC	TLS
1	Mathieu. Picardeau	France	117	4,751	40.61	5,969
2	Walter. Lilenbaum	Brazil	89	1,170	13.15	1704
3	Albert I. Ko	USA	62	3,943	63.60	4,074
4	Silvio A. Vasconcellos	Brazil	62	1729	27.89	3,777
5	Nobuo. Koizumi	Japan	55	938	17.05	1,175
6	Ana L.T.O. Nascimento	Brazil	53	1,382	26.08	3,978
7	Mitermayer G. Reis	USA	50	2,216	44.32	2,770
8	YungFu Chang	USA	47	1,249	26.57	2003
9	Adler. Ben	Austria	46	3,302	71.78	3,995
10	David A. Haake	USA	45	2,777	61.71	3,943

TC, Total citations; AAC, Average article citations; TLS, Total link strength.

### 3.5 Journal and co-cited journal analysis

Since 1993, leptospirosis research has been published across 955 academic journals. Using VOSViewer, co-occurrence relationships between journals (Figures 3I,J) and co-citation relationships were visualized (Figures 3K,L). Table 4 and Supplementary Table S2 list the top 10 journals and co-cited journals. “PLOS Neglected Tropical Diseases” ranks first with 166 articles, followed by “Journal of Wildlife Diseases” (126 articles) and “PLOS One” (123 articles). Among the top ten journals, three belong to the Q2 quartile, with “Journal of Clinical Microbiology” having the highest impact factor (IF: 6.10), followed by “PLOS Neglected Tropical Diseases” (IF: 3.40), highlighting their academic significance in leptospirosis research. Notably, most of these journals are based in the United States, reflecting the country’s dominance in the field, consistent with earlier findings on country/region and institutional contributions.

**Table 4:** Top 10 journals in terms of number of articles published.

Journal	Country	IF (2023)	JCR (2023)	Citations	Documents	AAC	TLS
PLOS neglected tropical diseases	USA	3.40	Q2	5,660	166	34.10	3,718
Journal of wildlife diseases	USA	1.10	Q4	2,543	126	20.18	743
PLOS one	USA	2.90	Q3	3,498	123	28.44	2,350
American journal of tropical medicine and hygiene	USA	1.90	Q4	2,716	111	24.47	1,264
Infection and immunity	USA	2.90	Q3	6,591	101	65.26	2,844
Veterinary microbiology	NETHERLANDS	2.40	Q2	3,174	78	40.69	1,612
Journal of clinical microbiology	USA	6.10	Q2	3,745	76	49.28	1,377
Veterinary record	ENGLAND	1.80	Q3	1,386	61	22.72	624
Epidemiology and infection	ENGLAND	2.50	Q4	1743	59	29.54	849
Journal of bacteriology	USA	2.70	Q3	2,736	59	46.37	755

AAC, Average article citations; TLS, Total link strength; IF, Impact Factor; JCR, Java Content Repository.

**Figure 4:**
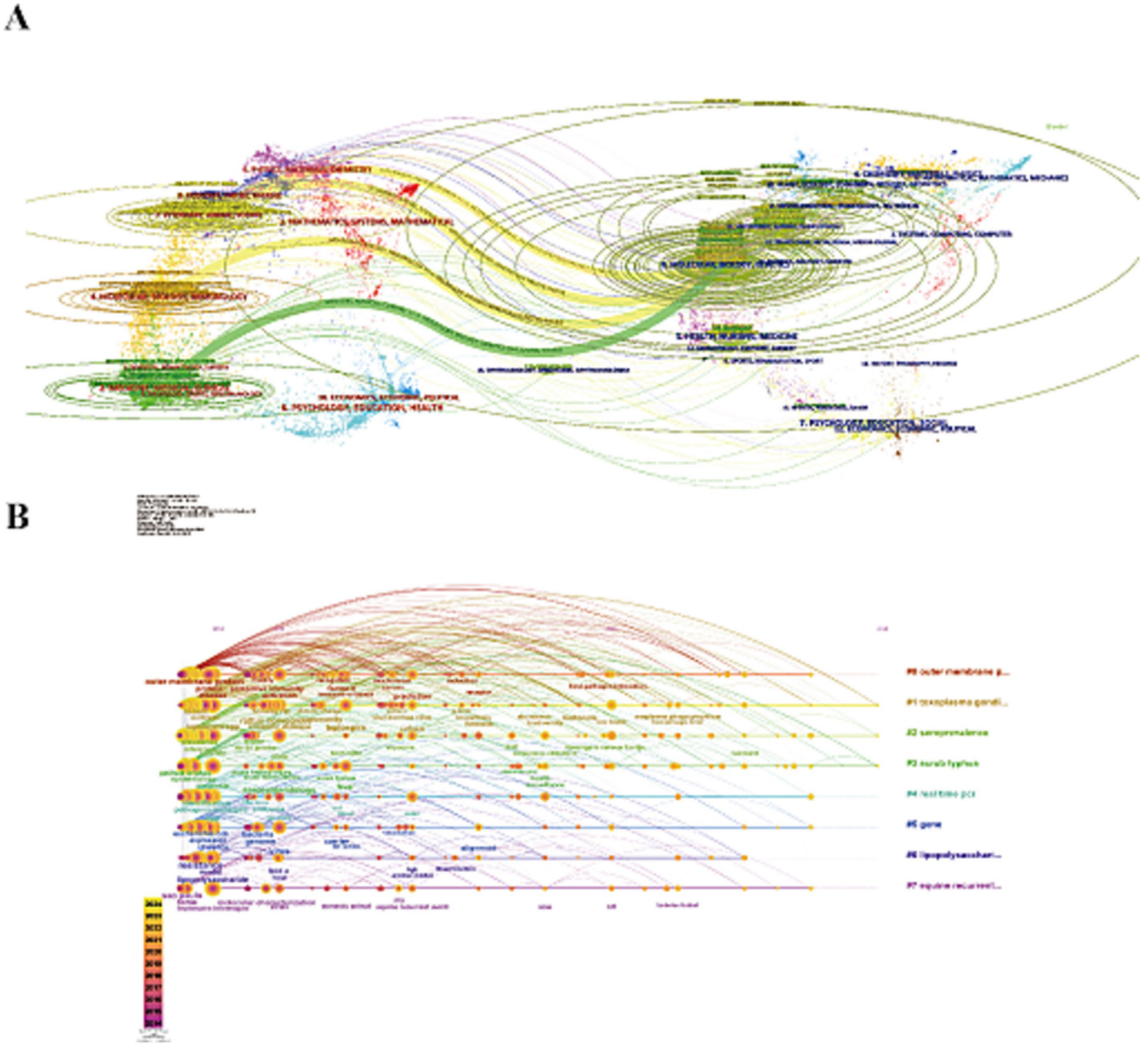
**(A)** Dual-map overlay of journals. The left nodes represent the included literature; the right represent the references in the literature. The labels represent the discipline. The link represents the cited path. **(B)** The timeline view of reference clusters by using CiteSpace software.

Subsequent CiteSpace analysis included dual-map overlays (Figure 4A), linking cited journals on the left with citing journals on the right. This provided a visual representation of citation relationships, topic distributions, and interdisciplinary movement patterns. Ellipses on the map represent the number of publications and author-to-publication ratios. Citation trajectories, depicted by thicker *Z*-scores, indicate stronger citation links. Four main citation trajectories were identified: three yellow and one green. The first yellow trajectory, focused on veterinary and animal science, was influenced by veterinary, animal, and parasitology research (*Z* = 2.54, *f* = 3,567). The second yellow trajectory, driven by molecular biology and genetics, had a *Z*-score of 4.71 and 6,275 publications. The third yellow trajectory, which intersected molecular biology and immunology, had a *Z*-score of 6.99 with 9,133 publications. The green trajectory, representing medicine and clinical fields, was influenced by molecular biology and genetics (*Z* = 4.13, *f* = 5,549).

### 3.6 Cluster analysis and keywords

**Figure 5:**
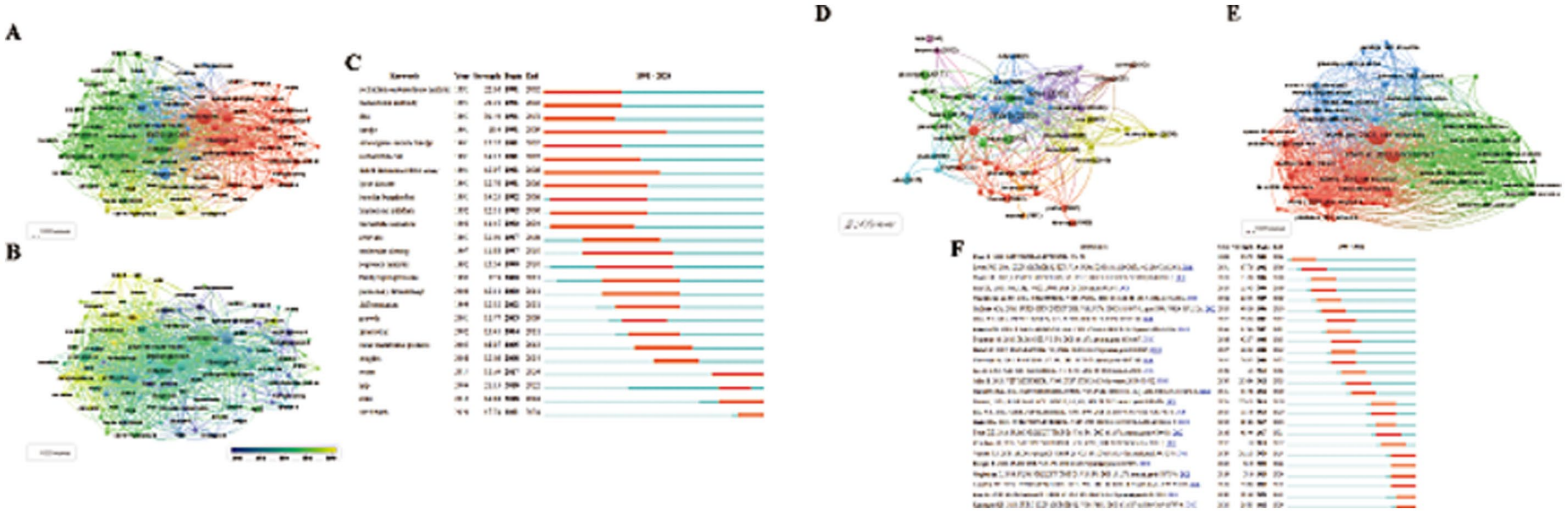
**(A,B)** Visual maps of keywords generated by VOSviewer. **(A)** Different color groups in the figure represent the relationship between keywords. **(B)** Different color groups in the graph represent the time points at which each keyword appears. **(C)** The top 25 keywords with the strong citation bursts. **(D,E)** Visual maps of highly cited References and co-cited References generated by VOSviewer (Different color groups in the figure represent the relationship between highly cited References and co-cited References). **(C)** The top 25 References with the strong citation bursts.

Using VOSviewer, 119 keywords appearing more than 50 times were identified, resulting in four distinct clusters (Figures 5A,B; Table 5). Core terms include “leptospirosis, ““*Leptospira*, ““diagnosis,” and “sepsis.” Cluster 1 (Red) consists of 45 terms, such as “activation,” “antigen,” “bacteria,” “binding,” “*Borrelia burgdorferi*,” “cells,” “*Escherichia coli*,” and “expression,” focusing primarily on the etiology and pathogenic mechanisms of *Leptospira*. Cluster 2 (Green) includes 42 terms related to the transmission routes of *Leptospira* in animals, such as “abortion,” “animals,” “antibodies,” “bovine,” “bovine leptospirosis,” and “Brazil.” Cluster 3 (Blue) features 19 terms like “assist,” “diagnosis,” “differentiation,” “DNA,” and “epidemic,” highlighting the role of DNA technology in diagnosing *Leptospira*. Cluster 4 (Yellow) comprises 13 terms, including “canine leptospirosis,” “disease,” and “ELISA,” focusing on various detection methods.

**Table 5:** Top 10 keywords in terms of number of articles issued.

Rank	Keyword	Occurrences	TLS
1	Leptospirosis	1,424	5,474
2	Leptospira	1,086	3,984
3	Infection	739	3,292
4	Interrogans	612	2,809
5	Diagnosis	535	2,458
6	Prevalence	502	2,624
7	Antibodies	484	2,471
8	Identification	435	1719
9	Cattle	378	1781
10	Seroprevalence	341	1896

TLS, Total link strength.

### 3.7 Keyword citation burst analysis and trend topics

Keywords with sudden citation bursts indicate emerging areas of research. The top 25 keywords exhibiting the strongest citation bursts are shown in Figure 5C, with the red line marking the duration of the burst. The research hotspots can be categorized into three stages: the early stage (before 2004) included keywords like “restriction endonuclease analysis,” “monoclonal antibody,” “DNA,” “Hardjo,” “*Interrogans serovar Hardjo*,” “*Escherichia coli*,” “linked immunosorbent assay,” “Lyme disease,” “*Borrelia burgdorferi* treponema,” and “pallidum nucleotide sequence.” The mid-term period (2005–2017) highlighted keywords such as “outer membrane proteins,” “insights,” and “water.” The most recent stage (2018-present) saw citation bursts for “spp.,” “state,” and “One Health.” The analysis of keyword bursts reveals evolving research trends and reflects the dynamic nature of the field, helping researchers quickly identify emerging research directions. Figure 4B demonstrates that from 2014 to 2024, research in the seven clusters continued to intensify, with the following key keywords showing sustained attention: "outer membrane protein," "Toxoplasma gondii," "*seroprevalence*," "scrub typhus," "real-time PCR," "gene," "lipopolysaccharide," and "equine recurrent uveitis".

### 3.8 Analysis of highly cited studies and co-cited studies

The top 10 highly cited studies on *Leptospira* are presented in Figures 5D,E. Noteworthy recent papers from the co-cited literature salience chart include Paul N. Levett’s seminal work, “Leptospirosis,” published in Clinical Microbiology Reviews in 2001, which has accumulated 3,988 citations and a TLS of 60. The second most cited study is Ajay R. Bharti’s “Leptospirosis: a zoonotic disease of global importance,” published in Lancet Infectious Diseases in 2003, with 2,828 citations and a TLS of 19. Ben Adler’s article, *Leptospira* and leptospirosis, ranks third, published in Veterinary Microbiology in 2010, with a TLS of 100 and 1983 citations.

CiteSpace analysis of citation bursts, which identifies articles with the strongest citation bursts, provides insight into future research directions. Figure 5F highlights the top 25 references with the most significant citation bursts, with the burst duration marked in red. The study Global Morbidity and Mortality of Leptospirosis: A Systematic Review has the highest burst intensity (intensity = 126.73), shedding light on the global incidence and mortality of leptospirosis. The review emphasizes leptospirosis as a leading zoonotic disease, with mortality rates comparable to or exceeding those of hemorrhagic fever, particularly in regions with limited research and statistical resources. Ben Adler’s 2010 paper, *Leptospira* and leptospirosis, ranks second in burst intensity (intensity = 110.84) and provides a comprehensive analysis of *Leptospira*’s biology and pathogenesis, forecasting significant progress through the interaction of the pathogen with its mammalian hosts and the environment. Additionally, two co-cited references emerged during the recent outbreak phase. The first is Leptospirosis: A Neglected Tropical Zoonotic Infection of Public Health Importance – An Updated Review, published in European Journal of Clinical Microbiology & Infectious Diseases by Krishnan Baby Karpagam et al. This review offers a detailed introduction to leptospirosis, covering its occurrence, transmission, clinical manifestations, diagnosis, treatment, preventive measures, and future prospects. The second is Kenneth Boey’s 2019 study, *Leptospira* Infection in Rats: A Literature Review of Global Pandemic and Distribution, published in PLOS Neglected Tropical Diseases. This paper summarizes global data on *Leptospira* infection in rats, comparing prevalence rates, geographic distribution, detection methods, and serogroup diversity, providing essential insights for global *Leptospira* research. These authoritative reviews are critical for guiding further research and addressing key gaps in understanding the disease’s global impact.

## 4 Discussion

To accurately predict research hotspots and identify future directions in *Leptospira* research, a comprehensive bibliometric analysis of global literature from 1967 to 2024 was performed using CiteSpace and VOSViewer software. The United States, Brazil, and France have been key contributors to *Leptospira* research, with the United States and Brazil leading in publications. Numerous institutions in these countries have conducted extensive studies, benefiting from a strong commitment to *Leptospira* research. The publications from scholars in these nations, along with those from France, are highly cited, reflecting close international collaboration and high academic influence in the field. In terms of research institutions, TLS values with medical institutions in Brazil, the United States, and France are notably high, indicating significant exchanges and cooperation in the field. This cross-national and institutional synergy is pivotal for advancing research. Moving forward, China and other countries should foster the establishment of international and multi-regional research centers, ensuring stable, multi-center collaborative research and striving to produce high-quality, highly cited publications to enhance academic impact. Regarding author contributions, Albert I. Ko, David A. Haake, and Ben. Adler have emerged as prominent experts, consistently ranking high in citation volume and TLS for both citing and co-referencing literature. Their work has been instrumental in advancing *Leptospira* research, and their research teams represent valuable potential collaborators for future projects.

Among the top 10 journals, PLOS Neglected Tropical Diseases focuses on infectious diseases, critical care medicine, and related fields (José María et al., 2021). As the journal with the highest number of published articles, it is regarded as a leading international platform for tropical medicine research. Published by the Public Library of Science, it covers pathology, epidemiology, prevention, treatment, control of neglected tropical diseases, and associated public policies. The journal emphasizes human disease research in the fields of translational medicine and epidemiology, regularly publishing original research articles, brief communications, reviews, laboratory studies, and cutting-edge scientific developments in tropical medicine. Other journals in the top 10 also exert considerable influence on *Leptospira* research. In conclusion, these top journals will remain essential for accessing pivotal and groundbreaking findings in the field of *Leptospira*. By analyzing these key journals, researchers can identify the most suitable outlets for publishing their work on *Leptospira* and target high-impact journals for disseminating their significant results. The overlay map of journals and disciplines in this study reveals that leptospirosis research intersects with basic sciences, clinical practice, nursing, and social sciences, underscoring the importance of interdisciplinary and collaborative research in this field.

As a significant zoonotic pathogen, *Leptospira* has garnered considerable attention in recent years. Our quantitative analysis of *Leptospira*-related literature revealed substantial shifts in research directions and emerging hotspots over time. By analyzing the top 10 keywords, temporal overlay visualizations, and emerging trends, three major phases in *Leptospira* research were identified. Key developments and trends were discussed in relation to burst keywords at different time points, as shown in Figure 5C.

### 4.1 Early stage (before 2004): basic research and molecular biology exploration

Before 2004, *Leptospira* research was primarily focused on understanding its basic biological characteristics and immunological properties. Keywords such as “restriction endonuclease analysis,” “monoclonal antibody,” “DNA,” “*hardjo*,” “serovar., “*Escherichia coli*, “linked immune assay,” “Lyme disease,” *Borrelia burgdorferi*, *Treponema pallidum*, and “nucleotide sequence” capture the research priorities of this period. During this phase, restriction enzyme analysis emerged as a pivotal tool for studying *Leptospira*’s genomic structure and diversity. Notable studies from this era include William A. Ellis’s 1991 work, which utilized 20 types of restriction endonucleases to analyze the DNA of various *Leptospira* strains, demonstrating the utility of this method as a classification tool for identifying pig isolates of the southern *Leptospira* serogroup (Ellis et al., 1991). In 1994, M. L. Savio highlighted the role of polymerase chain reaction (PCR) combined with DNA amplification and restriction endonuclease analysis in detecting and identifying *Leptospira* serum samples (Savio et al., 1994). Furthermore, in 1996, Carole A. Bolin et al. employed DNA restriction endonuclease analysis to investigate restriction fragment length polymorphism, establishing a correlation between this polymorphism and the host animal origin (Bolin and Zuerner, 1996). This analytical approach significantly advanced epidemiological studies of leptospirosis, aiding in the understanding of genetic variation among *Leptospira* strains across regions and their transmission routes among hosts.

The development and application of monoclonal antibodies also played a pivotal role in the identification of *Leptospira* antigens, providing foundational tools for immune diagnostics and vaccine development. In 1983, Ben. Adler et al. first identified species-and genus-specific antigens of *Leptospira* using monoclonal antibodies and enzyme immunoassays (Adler and Faine, 1983b). Later that year, they utilized monoclonal antibodies to detect a Pomona serogroup-specific agglutination antigen in *Leptospira* (Adler and Faine, 1983a). Similarly, in 1987, Rance B. Lefebvre introduced a DNA probe for diagnosing and classifying the *Hardjo bovis* genotype, a North American bovine pathogen, which proved specific to this genotype and did not cross-react with the genomic DNA of other common *Leptospira* pathogens in North America (LeFebvre, 1987). Additional advances included the application of monoclonal antibodies in China in 1989 for isolating *Leptospira* serotypes, significantly enhancing diagnostic accuracy (Zhang et al., 1989). In 1991, John V. Hookey et al. used monoclonal antibodies and DNA restriction endonucleases to identify jaundice hemorrhagic strains of *Leptospira* from various reference laboratories (Hookey and Palmer, 1991). These studies collectively established monoclonal antibody usage as a key method for *Leptospira* identification, laying the groundwork for further research in immune diagnostics and vaccine development.

In 1993, M. L. Savio et al. employed recombinant probes and serovar-specific monoclonal antibodies to identify 31 *Leptospira* strains from cattle and pigs. This study marked a pioneering integration of immunological and genetic approaches, combining monoclonal antibody-based identification with genomic analysis to assess strain variability (Savio et al., 1993). The early 2000s saw further advancements in diagnostic techniques. In 2005, Junpen Suwimonteerabutr utilized a monoclonal antibody-based dot blot Enzyme-Linked ImmunoSorbent Assay (ELISA) to detect *Leptospira* infection in blood and urine samples. This method was compared with dark-field microscopy, microbial culture, and PCR, highlighting its effectiveness in detecting *Leptospira* in bovine urine samples (Junpen et al., 2005).

Research during this period also explored the similarities between *Leptospira* and other *spirochete* pathogens, such as *Borrelia burgdorferi* (*B. burgdorferi*) and *Treponema pallidum*, suggesting potential cross-immune responses and shared evolutionary pathways. For example, in 1994, James L. Coleman identified escape variants of *B. burgdorferi* and used monoclonal antibody selection to express a truncated form of OspB. The resulting mutations provided insight into immune evasion mechanisms, with implications for *Leptospira* diagnosis and vaccine development (Coleman et al., 1994). The investigation of immune escape mechanisms in *Leptospira* continued, focusing on live pathogenic spirochete populations. For instance, Antonella et al. (2004) found that live *Leptospira* and *T. pallidum* stimulated Kupffer cells *in vitro*, triggering cellular responses and varying levels of tumor necrosis factor-alpha release (Antonella et al., 2004). Researchers developing moderately infectious, non-pathogenic live vaccines for *Leptospira* also discovered that complement-mediated selection in tissue co-cultures provided an innovative method for isolating pathogenic spirochete populations from attenuated strains. In 2005, Ece Sen demonstrated that complement resistance and pathogenic phenotype could be used to isolate *B. burgdorferi* in co-culture systems supported by tissue feeder layers from LEW/N rat tibial joints. This work confirmed that animal passage of complement escape variants is essential for isolating helicoids from highly passaged, non-arthrogenic, and attenuated cultures (Ece and Ronald, 2003). These studies provided key insights into the immune escape mechanisms of *Leptospira*, laying the foundation for subsequent research on pathogenesis and immune response. In summary, this period represents a shift from basic microbiological studies to more applied research focused on the prevention and treatment of *Leptospira* infections. By exploring the function of outer membrane proteins, researchers not only revealed the molecular mechanisms underlying *Leptospira* pathogenicity but also laid the groundwork for the development of novel prevention and therapeutic strategies.

### 4.2 Mid-term stage (2005–2017): study on membrane protein and influence of environmental factors

From 2005 to 2017, research on *Leptospira* increasingly focused on the functional roles of outer membrane proteins and the organism’s environmental survival strategies. This shift in focus is reflected in keywords such as “outer membrane proteins,” “insights,” and “water.” During this period, outer membrane proteins were identified as key pathogenic determinants, influencing *Leptospira*’s interaction with host cells and its virulence. The adhesion and invasion of host mammalian cells *in vitro*—primarily observed in highly virulent strains—was found to be a key step in its pathogenicity. This adhesion, absent in cultured, attenuated, or saprophytic *Leptospira* strains, likely involves molecules secreted by the bacteria or present on their surface (Merien et al., 1997; Michele et al., 2002). Several surface proteins of *Leptospira* have been shown to bind to multiple extracellular matrix components *in vitro* (Stevenson et al., 2007; Merien et al., 2000). Cryo-electron microscopy revealed that highly virulent strains exhibit a significantly reduced number of protein particles on their outer membrane, with distinct protein and lipopolysaccharide (LPS) profiles compared to attenuated strains (David et al., 2008).

As with other *spirochetes*, the *Leptospira* genome contains a high number of lipoprotein genes. Genomic analysis of *L. interrogans* revealed approximately 145 lipoproteins, as well as several extracellular and outer membrane proteins (João et al., 2005; Wasna et al., 2008). Consistent with *Leptospira*’s ability to migrate through host tissues, its genome encodes a variety of hemolysins and proteases that likely contribute to this process. The *L. interrogans* genome contains nine genes encoding hemolysins, including the sphingomyelinase gene (Mathieu et al., 2008) and pore-forming protein gene (Seoung Hoon et al., 2001), absent in saprophytic *L. biflexa*. Additionally, *L. interrogans* possesses a microbial collagenase, implicated in host tissue degradation. However, very few proteins have been experimentally verified on the surface of hook bodies (Paul et al., 2004).

Current literature identifies 12 proteins as outer membrane components, including OmpL1 (Haake et al., 1993), LipL32 (Haake et al., 2000), LigB (James et al., 2003), and Loa22 (Paula et al., 2007). Loa22, exposed on the bacterial surface, is recognized by the serum of patients with leptospirosis (Marcia et al., 2005) and upregulated in acute infection models. While the binding of Loa22 to extracellular matrix components *in vitro* is relatively weak (Angela et al., 2006), LipL32, also known as Hap-1, a hemolysis-related protein (Lee et al., 2000), comprises 75% of the outer membrane proteome (Paul et al., 2002). The lipoproteins in pathogenic *Leptospira* are highly conserved (David et al., 2004), with homologs of LipL32 absent in *L. biflexa*. LipL32 has long been considered a potential virulence factor, with its expression in acute lethal infections higher than *in vitro* cultured *Leptospira* (Jarlath et al., 2006). Further advancing the understanding of *Leptospira*’s pathogenic mechanisms, high-molecular-weight proteins—LigA, LigB, and LigC—were identified as members of the bacterial immunoglobulin (BIg)-like protein superfamily (Nobuo and Haruo, 2004; Raghavan et al., 2002). These proteins, anchored on the outer membrane, contain 12 to 13 tandem repeat domains. Similar to LipL32, Lig proteins are found exclusively in pathogenic *Leptospira*. Recombinant Lig proteins bind to host extracellular matrix proteins, such as fibronectin, fibrinogen, collagen, and laminin (Choy et al., 2007; Lin and Chang, 2007). The repetitive domain of LigB also binds calcium, enhancing its adhesion to fibronectin (Lin et al., 2008). These proteins are upregulated under physiological osmotic pressure and are recognized by the serum of patients with leptospirosis (Julio et al., 2007; Srimanote et al., 2008). In addition to the aforementioned proteins, other outer membrane proteins, such as those from *Leptospira sero*var *Shermani*, have been shown to enhance extracellular matrix synthesis, suggesting a potential link between *Leptospira*-induced acute renal tubulointerstitial nephritis and renal tubulointerstitial fibrosis (Ya-Chung et al., 2006). James Matsunaga, in 2006, identified the novel surface-exposed lipoprotein LipL46, distinguishing it from the previously characterized P31 (LipL45). Through surface immunoprecipitation and whole-cell ELISA assays, his team demonstrated that LipL46 is displayed on the surface of *Leptospira* (James et al., 2006).

These proteins are pivotal in host cell adhesion and invasion, and they likely contribute to immune evasion. The extensive study of outer membrane proteins has revealed their potential as vaccine candidates, offering promising avenues for vaccine development. During this period, research also emphasized *Leptospira*’s remarkable persistence in aquatic environments, a survival capability influenced by factors such as temperature and pH (Emilie et al., 2020). Leptospirosis, widely distributed across the globe, exhibits a higher incidence in tropical regions compared to temperate ones. Outbreaks often correlate with floods and hurricanes, which wash *Leptospira* from urine-contaminated soil into water bodies (Smita Benjamin et al., 2010). Reports of leptospirosis outbreaks have been documented in Brazil, India, and other areas following heavy rainfall. *Leptospira* can be isolated from various water bodies, including ponds, rivers, lakes, puddles, dams, springs, ornamental fountains, sewage, farmland, and moist soil. Flood-induced rainwater runoff can create alkaline conditions that support the pathogen’s environmental survival. *Leptospira* can persist in freshwater and moist soil for extended periods, from weeks to years, particularly under slightly alkaline conditions (Andrew et al., 2018; Arnau et al., 2018).

While leptospirosis is predominantly a waterborne disease, the bacterial load in soil is often underestimated. Human infections typically occur through skin contact with contaminated water or soil, though transmission *via* ingestion, inhalation, animal bites, or human-to-human contact remains rare (Didier and Bernard, 2013; Krishna Kanchan and Usha, 2008). Risk factors for *Leptospira* infection include occupations such as agriculture, veterinary medicine, slaughterhouse work, fishing, and sewage treatment. Additionally, individuals traveling to endemic areas, participating in freshwater sports, or engaged in disaster relief are at increased risk (Desai et al., 2016; Tippawan et al., 2018). Urban areas with rodent infestations, inadequate sanitation, and poor drainage systems are also at higher risk for leptospirosis outbreaks (Federico et al., 2014; José et al., 2016; Norlan de Jesus et al., 2017). These findings not only enhance our understanding of *Leptospira*’s ecological behavior but also underscore the critical need for effective water pollution control to mitigate public health risks.

### 4.3 Latest stage (2018–present): interdisciplinary integration and one health strategy

Since 2018, research on *Leptospira* has entered a new phase, driven largely by advancements in molecular biology. Current efforts are focused on elucidating molecular mechanisms and implementing precision medicine strategies. While further clarification of research trends is ongoing, emerging keywords highlight areas such as vaccine development, gene editing technologies, antimicrobial resistance, and novel diagnostic approaches. In vaccine development, studies have concentrated on outer membrane proteins of *Leptospira*, with initial findings indicating promising immune protection. DNA vaccines, a relatively recent advancement, have attracted significant attention due to their ability to sustain long-term expression of exogenous proteins in animal hosts, leading to prolonged antigen exposure. These vaccines offer several advantages, including cost-effective scalability, enhanced stability, and the capacity to encode multiple genes in a single construct (Marcelle Moura et al., 2017; Yingying et al., 2014). Furthermore, DNA vaccines can elicit both cellular and humoral immune responses, particularly when administered using prime-boost strategies. However, challenges persist, including relatively low immunogenicity in human clinical trials and potential tolerance development following prolonged antigen exposure (Garba and Abdul Rani, 2018).

To overcome these challenges, researchers are focusing on DNA vaccines targeting highly conserved proteins in pathogenic *Leptospira*, which are absent in saprophytic strains. One such protein, LipL32, is the most abundant in pathogenic strains and has been extensively studied as a potential antigen due to its strong immunogenicity in animal models (Guerreiro et al., 2001; Xiang-Yan et al., 2005). The first DNA-based *Leptospira* vaccine utilized the LipL32 gene, delivered *via* the mammalian expression vector pCDNA3.1. This vaccine conferred significant protection to mice against a lethal challenge with *L. interrogans serovar Canicola*, resulting in a 60% survival rate, compared to just 35% in the control group (Branger et al., 2005). Another DNA vaccine, using plasmid pTarget/LipL32, was evaluated alongside recombinant subunit vaccines and live recombinant BCG vaccines. All three vaccine platforms induced robust humoral immune responses, with antibodies capable of recognizing native proteins on the intact membrane of *Leptospira* (Fabiana Kömmling et al., 2007a).

Feng et al. further evaluated DNA vaccine efficacy using a prime-boost strategy that combined recombinant LipL32 with LipL41 and OmpL1 (LipL32-41-OmpL1). Mice immunized with these DNA constructs exhibited significantly higher levels of IL-2 and IFN-*γ* compared to those vaccinated with homologous proteins, indicating the enhanced immune responses facilitated by DNA vaccination. The prime-boost regimen also induced a more robust antibody response and a more pronounced cytokine profile than DNA vaccination alone (Feng et al., 2009). OmpL37, another surface-exposed protein in *Leptospira*, demonstrated a higher adhesion affinity for elastin tissue than other outer membrane proteins, suggesting its critical role in the infection process (Marija et al., 2010; Mariko et al., 2012). Studies investigating the protective immune response of an OmpL37 DNA vaccine using a prime-boost approach found no significant protection in groups receiving only the DNA vaccine, although notable IgG responses were observed in the prime-boost group (Thaís Larré et al., 2015).

The live vaccine strategy relies on attenuated self-replicating microorganisms with immune-stimulating properties, such as BCG (Fabiana Kömmling et al., 2007b) or adenovirus (Branger et al., 2001), to deliver exogenous antigens from pathogens. Seixas and colleagues employed various methods to screen for the immune-protective activity of LipL32, including a DNA vaccine in the pTarget vector, recombinant protein expression in *E. coli* using the pAE expression vector, and live recombinant BCG. These constructs were used to immunize mice, and the evaluation of humoral immune responses revealed that the rLipL32 antibody titer was the highest, while the LipL32 rBCG group showed a steady increase in antibodies (Fabiana Kömmling et al., 2007a). Although not directly evaluated, it was anticipated that rBCG would elicit a strong cellular immune response against heterologous antigens. Growth inhibition of *Leptospira* was observed *in vitro* in the presence of an anti-LipL32 monoclonal antibody, further highlighting LipL32’s immunogenicity. Seixas and colleagues also explored the capacity of rBCG to express LipL32 as an antigen against leptospirosis (Fabiana Kömmling et al., 2007b), revealing that immunization with various rBCG/LipL32 constructs promoted serum conversion against LipL32, with higher antibody titers than the control group. In a hamster model of leptospirosis, however, inconsistent data on the protective efficacy of rBCG/LipL32 immunization were observed. In subsequent work, the team developed a recombinant chimeric multi-epitope vaccine, r4R, consisting of four repeats of T and B cell binding epitopes from six identical proteins (OmpL1, LipL32, and LipL21). This vaccine was tested for its ability to induce protective immune responses in guinea pigs. Compared to the PBS control group, r4R immunization resulted in increased survival rates and reduced renal colonization following infection with pathogenic *Leptospira* (Xu'ai et al., 2016).

Gene-editing technologies, such as CRISPR/Cas9, have emerged as powerful tools for investigating *Leptospira* gene function and drug resistance mechanisms. Sirawit Jirawannaporn’s team developed an RPA CRISPR/Cas12a detection platform to identify the LipL32 gene in pathogenic *Leptospira* strains. This platform demonstrated impressive sensitivity (85.2%), specificity (100%), and accuracy (92.7%) in a study involving 110 patients across 15 hospitals in Thailand. In addition, a lateral flow detection test was developed to accurately distinguish known positive and negative clinical samples, highlighting the potential of RPA CRISPR/Cas12a technology in enhancing the speed and precision of spirochete diagnosis, particularly in resource-limited settings (Sirawit et al., 2022). Further research in 2023 confirmed the platform’s high specificity and accuracy, positioning it as a promising tool for early detection and effective treatment, especially in rural healthcare settings (Sirawit et al., 2023). These advancements in *Leptospira* research, particularly in vaccine development and diagnostic methods, reflect a comprehensive approach to combating this global public health challenge. Future efforts should continue to incorporate interdisciplinary methodologies to refine diagnostic accuracy and develop robust therapeutic strategies.

In terms of diagnosis, rapid detection methods for molecular markers—such as those based on the microscopic agglutination test (MAT), PCR, and next-generation sequencing (NGS)—have substantially improved diagnostic efficiency for *Leptospira* infections. MAT remains a standard diagnostic tool, offering high sensitivity during the early stages of infection. However, its sensitivity diminishes as antibody titers decrease over time. A positive diagnosis is typically confirmed when the titer value is ≥400, or the paired serum titer value is ≥100 (Jane et al., 2022). While MAT is considered the ‘gold standard, it has limitations, including lack of specificity for IgM or IgG antibodies, and the requirement for well-trained technicians to interpret results and maintain live *Leptospira* cultures in enriched EMJH medium (Krishna Kanchan and Usha, 2008).

PCR has become a critical molecular tool for *Leptospira* detection due to its high specificity and sensitivity, enabling the identification of even trace amounts of bacterial nucleic acid. C. Gravekamp et al. demonstrated that PCR could achieve 100% specificity under controlled conditions (Gravekamp et al., 1993). Kate Woods et al. later found that quantitative PCR (qPCR) performed upon patient admission is not only a reliable and rapid diagnostic method but also outperforms both MAT and culture techniques. This underscores its significance in clinical and epidemiological investigations of this neglected disease (Woods et al., 2017). Other PCR techniques, such as nested PCR and arbitrary primer PCR, are also employed for *Leptospira* detection (Senaka et al., 2015). However, PCR is not without its challenges, including the need for sophisticated laboratory setups, the risk of contamination, potential for false positives, and the requirement for skilled personnel and substantial financial investment. Real-time PCR (RT-PCR) has gained prominence due to its ability to deliver specific results quickly by monitoring DNA amplification rates. This method is capable of quantifying bacterial DNA load, providing valuable insights into infection severity. The LipL32 gene serves as the primary target for qPCR-based identification, known for its high specificity and sensitivity in the early stages of leptospirosis diagnosis (Daša et al., 2020). However, qPCR requires specialized, expensive equipment, making it impractical for low-resource or basic laboratory settings. To improve sensitivity and specificity across all stages of infection, various serological and molecular techniques have been developed. One such approach is immune capture polymerase chain reaction (IC-PCR), which combines ELISA with PCR. In 2018, Balassiano et al. introduced IC-PCR, focusing on serological rather than genotypic traits, thus offering a novel tool for early diagnosis and valuable epidemiological surveillance by characterizing infected sera or serum groups (Ilana Teruszkin et al., 2012).

Recently, metagenomic next-generation sequencing (mNGS) has gained attention for *Leptospira* detection, though it has primarily been documented in case reports. For example, Michael R. Wilson et al. reported the case of a 14-year-old boy with severe combined immunodeficiency, where second-generation sequencing of cerebrospinal fluid confirmed *Leptospira* infection, identifying *Leptospira santarosai* as the causative pathogen. This marked the first instance of *Leptospira* being detected *via* next-generation sequencing (Wilson et al., 2014). Another case involved a 75-year-old male diagnosed with pulmonary tuberculosis complicated by leptospirosis. Following delayed serological testing, mNGS of cerebrospinal fluid, urine, plasma, and sputum samples revealed both *Mycobacterium tuberculosis* complex and *Leptospira*, illustrating how mNGS can complement traditional diagnostic methods by providing comprehensive pathogen screening (Yunzhen et al., 2023). Ji et al. described the case of a 40-year-old pig farmer with severe pulmonary hemorrhagic leptospirosis and multiple organ failure, who was diagnosed with leptospirosis through mNGS, enabling timely antibiotic therapy adjustments. The patient recovered following intensive care unit treatment and extracorporeal membrane oxygenation support (Ji et al., 2023). As NGS technology continues to evolve, whole-genome sequencing has become increasingly integral to leptospirosis research. In 2014, Chou et al. completed the first whole-genome sequencing of *Leptospira santarosai sero*var *Shermani*, analyzing its transcriptional response and providing foundational insights into the pathogenic mechanisms of *Leptospira*. This work has helped inform the development of preventive and therapeutic strategies (Li-Fang et al., 2015). Similarly, Sérgio Jorge et al. employed next-generation sequencing to obtain the complete genome of a virulent *Leptospira* strain isolated from southern Brazil, enhancing understanding of the genetic characteristics of local isolates and aiding the identification of potential diagnostic and vaccine targets (Sérgio et al., 2017). With rapid advancements in molecular techniques, a variety of other methods have emerged, including DNA–DNA hybridization (Priscyla et al., 2023), restriction endonuclease analysis (REA) (Arent et al., 2017), random amplified polymorphic DNA (RAPD) fingerprinting (Rafael et al., 2019), and pulsed-field gel electrophoresis (PFGE) (Pejvak et al., 2024). These sophisticated methods, largely utilized in advanced laboratories in developed countries, have significantly expanded our understanding of genetic variation and genomic diversity within *Leptospira*.

The growing issue of antimicrobial resistance has become a major concern, particularly as *Leptospira* shows increasing resistance to traditional antibiotics, presenting new challenges for public health strategies. Early initiation of antibacterial therapy is crucial in preventing the progression of leptospirosis to more severe stages. For patients with severe leptospirosis requiring hospitalization, treatment typically involves intravenous administration of penicillin (1.5 million units every 6 h), ampicillin (0.5–1 gram every 6 h), ceftriaxone (1 gram every 24 h), or cefotaxime (1 gram every 6 h). In contrast, adult outpatients in the early stages of the disease are generally prescribed doxycycline (100 milligrams orally, twice daily) or azithromycin (500 milligrams orally, once daily). Pregnant women and children can also be treated with azithromycin or amoxicillin, with dosage adjustments based on body weight. These treatment guidelines are informed by *in vitro* susceptibility data, animal studies, and clinical experience, including a randomized, placebo-controlled, double-blind study demonstrating that doxycycline shortens the course of leptospirosis by two days and alleviates symptoms such as fever, discomfort, headache, and myalgia (Hospenthal and Murray, 2003; McClain et al., 1984). Additionally, doxycycline therapy can prevent the shedding of *Leptospira* in urine.

Despite the extensive research on *in vitro* antibiotic screening for *Leptospira*, studies on the *in vivo* efficacy of these treatments remain limited. Some research has even suggested that while antibiotics may show antibacterial effects *in vitro*, subtherapeutic dosing *in vivo* can worsen the clinical course of leptospirosis. Zhang et al. reported that treating golden hamsters with subtherapeutic doses of ciprofloxacin and norfloxacin increased mortality and aggravated pathological damage to the kidneys, liver, and lungs (Wenlong et al., 2014). Furthermore, although doxycycline demonstrates higher *in vitro* antibacterial potency compared to penicillin, *in vivo* studies have indicated its superior therapeutic effect, likely due to its immunomodulatory properties (Henrique et al., 2021). In late-stage leptospirosis, antibiotic administration becomes less effective. Jin et al. combined antibiotics with polyclonal antibodies, increasing the survival rate of golden hamsters infected with lethal leptospirosis to 50% (Xuemin et al., 2016), suggesting that a combination of antibiotics and antibody therapy could benefit patients with severe leptospirosis.

Antibiotics also play a significant role in influencing the prevalence of leptospirosis, particularly within the aquaculture industry. Pigs and mice are recognized as primary reservoirs for *Leptospira*, with *L. interrogans sero*var *Pomona* being common in pigs and *Leptospira serovar Lai* in mice. Interestingly, in recent years, the incidence of serovar Pomona in both humans and pigs has declined, while serovar Lai has become the dominant strain in human leptospirosis cases. A study found that adding oxytetracycline to pig feed effectively eliminated *Leptospira* infection at an early stage, leading to a reduction in the yearly incidence rate and a decreased risk of transmission to humans (Weilin et al., 2014). Recent research has also focused on utilizing immune modulators that target various host responses to enhance resistance to *Leptospira* infection. For example, Cao’s research group demonstrated protective effects in hamster models when pattern recognition receptor (PRR) agonists were administered during or one day before infection (Maria et al., 2017). Studies have shown that early expression of TLR2 occurs in tolerant Balb/c mice, whereas delayed expression is observed in more susceptible hamster models (Wenlong et al., 2016). This aligns with an earlier study by Matsui et al., which suggested that early activation of inflammatory mediators provides protection in *Leptospira*-infected mice, while a delayed response in hamsters may lead to a cytokine storm (Mariko et al., 2011). Co-injection of *L. interrogans serovar* Autumnalis and the synthetic TLR2 agonist Pam3cysSK4 in golden hamsters alleviated acute leptospirosis, reduced *Leptospira* load after three weeks, and mitigated pathological changes in organs (Wenlong et al., 2016). Furthermore, administering crude *E. coli* LPS to hamsters infected with *Leptospira* showed an increase in effectiveness, correlating with enhanced inflammation levels (Wenlong et al., 2020). Interestingly, co-injection of *β*-glucan (a fungal cell wall component that agonizes the Dectin-1 receptor and synergizes with TLR2) one day before infection also increased early inflammation and improved survival rates (Jiaqi et al., 2019).

Using *Lactobacillus plantarum* as a platform to express *Leptospira* proteins has shown unexpected protective effects in immune-sensitive C3H/HeJ mice when administered orally. After six weeks of intermittent gavage treatment, *L. plantarum* provided protection in these sensitive model mice (Hari-Hara et al., 2017). Although *L. plantarum* did not prevent kidney colonization, it reduced inflammation and fibrosis, suggesting the concept of ‘innate immune memory’ or ‘trained immunity, ‘where initial infection triggers metabolic and epigenetic reprogramming of macrophages and natural killer cells, leading to a heightened response to subsequent challenges. Further investigation by the team revealed that two intraperitoneal injections of CL429, a TLR2-NOD2 dual agonist, replicated the protective and anti-inflammatory effects observed in oral *L. plantarum* administration in viral lung infection models (Tyler et al., 2016). These results confirmed that CL429 pretreatment induces an innate memory effect, independent of B and T cells in the peritoneal cavity, as well as natural killer cells from distant sites like the bone marrow and spleen (Ignacio et al., 2019).

In conclusion, significant progress has been made in *Leptospira* research over recent decades. From early genomic and antigen characterization studies to mid-term investigations into the functions of outer membrane proteins and environmental survivability, and now to advanced explorations of molecular mechanisms and diagnostic technologies, the study of *Leptospira* is a dynamic and continuously evolving field. Future studies should continue integrating interdisciplinary approaches to develop more precise diagnostic tools and therapeutic strategies, addressing the global public health challenges posed by *Leptospira*.

## 5 Advantages and limitations

This study is the first to analyze the evolution and research trends of *Leptospira* using bibliometric methods. While bibliometric analysis offers a comprehensive and objective overview, it does come with certain limitations. Firstly, our study relied solely on publications from the WOSCC database, which may not encompass all relevant research on *Leptospira*. Secondly, we focused exclusively on English-language publications, introducing a potential linguistic bias by excluding research in other languages. Additionally, we did not assess the quality of the publications, treating high-quality and low-quality studies equally. This is an inherent limitation that should be acknowledged when interpreting the results.

## 6 Conclusion

To the best of our knowledge, this is the first comprehensive bibliometric analysis of research on *Leptospira*. The study of leptospirosis has gradually shifted from the early basic biological exploration to the in-depth study of molecular mechanisms, and is closely integrated with global health strategies at the latest stage. By analyzing the research hotspots at different stages, it can be seen that the research in this field has expanded from the early stage of pathogen identification and diagnostic tool development to understanding its pathogenic mechanism and environmental impact, and finally developed into an interdisciplinary integrated prevention and control strategy. Future research will continue to focus on global control of leptospirosis, especially in improving diagnostic capabilities, developing effective vaccines and reducing the risk of environmental transmission, contributing to reducing the public health burden of the disease.

## Data Availability

The datasets presented in this study can be found in online repositories. The names of the repository/repositories and accession number(s) can be found in the article/Supplementary material.
