# Supplementary material for: Trends and advances in Leptospira, a bibliometric analysis
Source: Front Microbiol. 2025 Jan 8;15:1514738. doi: 10.3389/fmicb.2024.1514738 (PMC11750782; doi:10.3389/fmicb.2024.1514738)
Supplement: Supplementary file 1 [file Supplementary_file_1.docx]

Supplementary Table 1 Top 10 co-cited authors

| Rank | Co-cited author | Country | TC | | TLS |  |
| --- | --- | --- | --- | --- | --- | --- |
| 1 | Paul N. Levett | Canada | 2127 | 30573 | |  |
| 2 | W. A. Ellis | Ireland | 1752 | 18529 | |  |
| 3 | Solly. Faine | Austria | 1588 | 21160 | |  |
| 4 | David A. Haake | USA | 1567 | 29525 | |  |
| 5 | Adler. Ben | Austria | 1567 | 22760 | |  |
| 6 | Ajay R. Bharti | USA | 977 | 13987 | |  |
| 7 | Federico. Costa | Brazil | 851 | 11645 | |  |
| 8 | Mathieu. Picardeau | France | 847 | 15299 | |  |
| 9 | Russell C. Johnson | USA | 774 | 9738 | |  |
| 10 | Albert I. Ko | USA | 707 | 12282 | |  |

Note: TC: Total citations; AAC: Average article citations; TLS: Total link strength
