# Supplementary material for: Trends and advances in Leptospira, a bibliometric analysis
Source: Front Microbiol. 2025 Jan 8;15:1514738. doi: 10.3389/fmicb.2024.1514738 (PMC11750782; doi:10.3389/fmicb.2024.1514738)
Supplement: Supplementary file 2 [file Supplementary_file_2.docx]

Supplementary Table 2 Top 10 co-cited journals in terms of number of articles issued

| Rank | | Journal | | Country | IF (2023) | JCR (2023) | Documents | TLS |
| --- | --- | --- | --- | --- | --- | --- | --- | --- |
| 1 | | Infection and immunity | USA | 2.90 | Q3 | 7793 | 243223 |  |
| 2 | | PLOS neglected tropical diseases | USA | 3.40 | Q2 | 5851 | 153662 |  |
| 3 | | Journal of clinical microbiology | USA | 6.10 | Q2 | 4354 | 100543 |  |
| 4 | | Journal of bacteriology | USA | 2.70 | Q3 | 4036 | 117243 |  |
| 5 | | PLOS one | USA | 2.90 | Q3 | 3784 | 116560 |  |
| 6 | | American journal of tropical medicine and hygiene | USA | 1.90 | Q4 | 3236 | 77822 |  |
| 7 | | Veterinary microbiology | NETHERLANDS | 2.40 | Q2 | 2843 | 69260 |  |
| 8 | | Veterinary record | ENGLAND | 1.80 | Q3 | 2527 | 46461 |  |
| 9 | | American journal of veterinary research | USA | 1.30 | Q3 | 2135 | 41430 |  |
| 10 | | Journal of wildlife diseases | USA | 1.10 | Q4 | 2037 | 33734 |  |

Note: AAC: Average article citations; TLS: Total link strength
