# Supplementary material for: Trends and advances in Leptospira, a bibliometric analysis
Source: Front Microbiol. 2025 Jan 8;15:1514738. doi: 10.3389/fmicb.2024.1514738 (PMC11750782; doi:10.3389/fmicb.2024.1514738)
Supplement: Supplementary file 3 [file Supplementary_file_3.docx]

Supplementary Table 3 Top 10 Co-cited References

| Rank | Title | Journals | Authors | Year | Citations | TLS |
| --- | --- | --- | --- | --- | --- | --- |
|  |  |  |  |  |  |  |
| 1 | Leptospirosis. | Clinical microbiology reviews | P N Levett | 2001 | 3988 | 60 |
| 2 | Leptospirosis: a zoonotic disease of global importance. | Lancet infectious diseases | Ajay R Bharti et al. | 2003 | 2828 | 19 |
| 3 | Leptospira and leptospirosis. | Veterinary microbiology | Ben Adler et al. | 2010 | 1983 | 100 |
| 4 | Global Morbidity and Mortality of Leptospirosis: A Systematic Review. | Plos neglected tropical diseases | Federico Costa et al. | 2015 | 1806 | 19 |
| 5 | Leptospira leptospir. | - | S Faine et al. | 1999 | 1510 | 73 |
| 6 | Leptospirosis in humans. | Current Topics in Microbiology and Immunology | David A. Haake et al. | 2015 | 1212 | 21 |
| 7 | Leptospira: the dawn of the molecular genetics era for an emerging zoonotic pathogen. | Nature reviews microbiology | Albert I. Ko et al. | 2009 | 1077 | 47 |
| 8 | Differentiation of pathogenic and saprophytic letospires. I. Growth at low temperatures. | Journal of Bacteriology | Russell C. Johnson et al. | 1967 | 995 | 6 |
| 9 | Unique physiological and pathogenic features of Leptospira interrogans revealed by whole-genome sequencing. | Nature | Shuang-Xi Ren et al. | 2003 | 801 | 26 |
| 10 | Leptospirosis. | Current opinion in infectious diseases | Alan J.A. McBride et al. | 2005 | 793 | 10 |
